# A Revised Kinetic Model for the Catalytic Partial Oxidation of Methane

**DOI:** 10.3390/e28060658

**Published:** 2026-06-09

**Authors:** Javier Jurado, Fernando Trejo, Jorge Ancheyta, Andrey Elyshev, Andrey Zagoruiko

**Affiliations:** 1 Instituto Politécnico Nacional, Centro de Investigación en Ciencia Aplicada y Tecnología Avanzada, Legaria 694, Col. Irrigación, Mexico City 11500, Mexico; jjurado1400@alumno.ipn.mx (J.J.); ftrejoz@ipn.mx (F.T.); 2 Escuela Superior de Ingeniería Química e Industrias Extractivas, Instituto Politécnico Nacional, Unidad Profesional Adolfo López Mateos, Zacatenco, Mexico City 07738, Mexico; 3 School of Natural Sciences, Tyumen State University, 15 a Perekopskaya St., Tyumen 625033, Russia; a.v.elyshev@utmn.ru (A.E.); zagor@catalysis.ru (A.Z.)

**Keywords:** methane partial oxidation, kinetic modeling, hydrogen production, methane steam reforming

## Abstract

The reaction scheme of the partial oxidation of methane is still currently undetermined for many catalysts due to a lack of accurate experimental data to develop kinetic studies. In this work, a kinetic modeling study of partial oxidation of methane was performed using experimental data from the literature. A kinetic model is proposed, and a new set of kinetic parameters was obtained in view of the failed reproduction of a model from the literature. The kinetic parameters of the proposed model were calculated and determined by using an optimization approach based on non-lineal parameter estimation, proper selection of initial values of parameters, and sensitivity and statistical analyses. Experimental data from the literature obtained in a packed-bed reactor with a Pt/Al_2_O_3_ catalyst at reaction temperatures of 465–574 °C were used to develop the kinetic model. Experimental data were accurately predicted by the proposed model with determination coefficient of 0.988.

## 1. Introduction

Partial oxidation of methane (POM) is one of the most important processes to produce syngas (H_2_ and CO). The great availability of natural gas and the increasing demand of hydrogen have aimed the research to optimize the production of syngas [1]. Natural gas, which is mainly composed by methane, is a promising resource in view of the decreasing world availability of petroleum [2]. Furthermore, the utilization of natural gas in the production of value-added chemicals will considerably increase its worldwide demand [3]. A wide production of syngas from methane can approach the environmental outlook to a net-zero carbon footprint in the current energy transition [4]. If the POM is substantially optimized, it may replace steam reforming (SR) for syngas production in the near future [5,6].

POM is a process that carries out the exothermic oxidation of methane. At low selectivity, the complete combustion of methane releases a high amount of heat in the process based on the total combustion of methane. At high selectivity, the oxidation of methane towards the production of hydrogen and carbon monoxide makes the process mildly exothermic based on the direct formation of methane to synthesis gas without formation of CO_2_ and H_2_O [7,8]. POM dominates the first zone of the catalyst bed while SR and water–gas shift (WGS) reactions become important in the remaining part of the bed [9]. The POM process is an attractive alternative since it avoids the need for large amounts of superheated steam and large reactors compared with SR, i.e., lower costs of operation [3,10,11]. Another advantage of direct partial oxidation is that it produces a H_2_/CO ratio of 2:1 [4,9]. POM can operate at gas hourly space velocity (GHSV) values from 10 to 100 times greater than SR. Some of the disadvantages of the POM process are the extreme temperatures, high space velocities, and high coke formation which is translated into a low controlled process, i.e., significant safety risk during operations [3,4,10]. The high exothermicity of the complete combustion of methane generates hot-spot temperature along the catalytic bed during the POM [7]. For this reason, catalysts with high stability and superior properties are required [4].

A complex reaction system consisting of multiple steps is carried out in POM [6]. A study of POM at temperatures below 1227 °C has been conducted to achieve a complex reaction scheme in which a set of kinetic equations are composed of hundreds of elementary steps [12]. Other non-complex reaction schemes and microkinetic models have also been proposed for kinetic modeling of POM [4]. However, the accuracy of all the kinetic models in the literature has been controversial [13]. Despite numerous studies, a unified mechanism for POM has not been fully accepted in the literature [14,15]. The POM reaction pathway can be determined by the catalyst composition, operating conditions, and the interactions between the promoter and support of the catalyst [7]. For all kinetic models, two reaction pathways are mainly used: (1) the total combustion of methane (R1) followed by reforming reactions (R3–R5) (indirect path) [16,17], and (2) the direct formation of methane to synthesis gas without formation of CO_2_ and H_2_O (R2) (direct path) [4,9,18]. These reactions are the following:

(1) $$\;\;\;\;R1:\;\;\;\;\;\;\;\;\;CH_{4}+2O_{2}\to CO_{2}+2H_{2}O\;\;\;\;\;\;\;\;\;\;\;\;\;\;\;\;\;\;\Delta H_{298K}=-801\;kJ/mol$$

(2) $$R2:\;\;\;\;\;\;\;\;\;CH_{4}+\frac{1}{2}O_{2}\to 2H_{2}+CO\;\;\;\;\;\;\;\;\;\;\;\;\;\;\;\;\;\;\;\;\Delta H_{298K}=-35\;kJ/mol$$

(3) $$\;\;\;\;R3:\;\;\;\;\;\;\;\;\;CH_{4}+H_{2}O⇄CO+{3H}_{2}\;\;\;\;\;\;\;\;\;\;\;\;\;\;\;\;\;\;\;\;\;\;\Delta H_{298K}=206.1\;kJ/mol$$

(4) $$\;\;\;\;R4:\;\;\;\;\;\;\;\;\;CH_{4}+2H_{2}O⇄CO_{2}+4H_{2}\;\;\;\;\;\;\;\;\;\;\;\;\;\;\;\;\;\;\Delta H_{298K}=165.0\;kJ/mol$$

(5) $$\;\;\;\;\;\;\;\;\;\;R5:\;\;\;\;\;\;\;\;\;CO+H_{2}O⇄CO_{2}+H_{2}\;\;\;\;\;\;\;\;\;\;\;\;\;\;\;\;\;\;\;\;\;\;\;\;\;\;\;\Delta H_{298K}=-41.15\;kJ/mol$$

A debate as to whether a dry reforming (DR) reaction occurs still remains [19]. The proof of the indirect route is the temperature hotspot located near the inlet of the reactor. While the main evidence of the direct route is the presence of syngas at various residence times with unreacted oxygen [4,9]. However, the hot spot could be eliminated if the POM mechanism occurs via the direct route [19]. The direct route of the POM pathway proposes the dissociation of methane into hydrogen and carbon. After that, hydrogen is desorbed and carbon oxidized by surface species [15].

Membrane reactors can improve POM performance, carrying out a more controlled combustion and a more spread-out heat release throughout the reactor since oxygen is supplied and separated from air along the reactor length [1]. High operating temperature increases methane conversion, H_2_ production and CO selectivity during POM performance [7], but it promotes CO_2_ production through the WGS reaction [4]. It has been found that the direct formation of CO is carried out over reduced Ni sites via a pyrolysis mechanism, while CO_2_ is produced by subsequent oxidation of CO [18]. At low temperatures, total oxidation reaction is dominating, but at high temperatures syngas production is increased [4]. At very short space times, Pt- and Rh-catalysts yield conversions far from the equilibrium conversion with a high syngas selectivity. A study based on Aspen HYSYS^®^ (Aspen Technology, Inc., Bedford, MA, USA) simulations concluded that higher pressures inhibit increasing hydrogen production by driving up the required operating temperatures [20].

Among other issues, self-sustained oscillations have been detected through X-ray diffraction techniques and mass spectrometry in the POM catalyst. The oscillations appear under oxygen-deficient conditions, provoking catalyst harm due to temperature peaks and the influence of the surface coverage with adsorbates [21]. Hence, POM catalysts require properties with high thermal stability and resistance to carbon deposition, especially with oxygen-permeable membrane reactors. POM catalysts are usually composed of a good variety of combinations of metal oxide supports (titanium dioxide, ferric oxide, alumina, silica, zirconium dioxide, lanthanum oxide) and metal promoters (Ni, Co, Cu, and Fe) [4,9]. Ni-based catalysts, also used for SR, offer good activity for POM to syngas [9,16]. Ni-based catalysts are low cost and have similar activity to noble metals. However, their drawback is their high tendency towards carbon deposition [4,10]. Most of the POM catalysts seem to promote the indirect reaction pathway at intermediate temperatures (700–800 °C) [18]. Noble metals offer exceptional activity, selectivity and resistant properties during POM performance compared with non-noble metals [4]. Rh-supported catalysts have performed the best results compared with the other noble and non-noble metals catalysts [5]. Although noble metal catalysts gave the best POM results, carbon deposition is unavoidable [6]. Some modifications to the POM catalysts have enhanced their performance. For instance, CeO_2_ support improves Ni site dispersion and stabilization, furthermore, its high oxygen storage capacity effectively reduced carbon deposition [14]. In another study, supports such as TiO_2_ or CeO_2_-ZrO_2_ act as oxygen storage and reoxidized carbon deposits, improving the catalyst stability [7]. The promoter of the total oxidation of methane (R1) of the Ni/MgO catalyst carries out syngas production by the indirect pathway route [15]. Studies on methane reactivity over Rh/SiO_2_ catalysts have reported that CO is formed without oxygen in the feed, which suggests that oxygen in the catalyst support can oxidize the carbon dissociated from methane, following the direct reaction pathway (pyrolysis) [14]. It is reported that, after oxygen adsorption, O_2_ back-spillover affects the stability of M-O bonds in metal sites converting them into metal phase [6]. The POM direct route was detected over a reduced Ni/Al_2_O_3_ catalyst, i.e., dissociation mechanism, while methane is oxidized to carbon dioxide and water over NiO/Al_2_O_3_ catalyst, and—at critical temperature—NiO is reduced to Ni^0^ again. CO selectivity is reduced over NiO/Al_2_O_3_ [5].

The optimization of chemical processes requires exhaust studies of kinetic and reactor modeling. The consideration of physicochemical and technological parameters solves many problems in the scaling-up of the processes from bench, to pilot, to full-scale industrial units. The main challenges found in heterogeneous reactor modeling are the selection of suitable kinetic models, algorithm and parameters to solve complex differential equations, and nonlinear optimization methods for the solution of the model [22]. In other words, if the kinetic model is not properly selected and the corresponding kinetic parameters (reaction rate coefficients, activation energies) are not the optimal ones, the modeling and further design of the reactor can provide inaccurate responses to the effects of reaction conditions. A POM kinetic model for use in a wide range of operating conditions (temperature, GHSV, different inlet compositions) has not been reported in the literature [6]. The calculation of pre-exponential factors for the steps of both direct or indirect mechanisms has been reported to be a tough task [23]. Although power-law models can simplify some POM predictions, they do not describe the underlying complex mechanism [6]. The development of more reliable and physically consistent kinetic and reactor models might give insights into the complexity of POM experiments. However, the necessary experimental investigations about different feed gas compositions (CH_4_, O_2_, CO_2_, CO, H_2_, H_2_O) are also scarce [9]. The significance of this work is its aim to describe the POM section of methane ATR, considering the abrupt changes in temperature of more than 100 °C taking place in the catalyst bed near the inlet of the reactor [11,24,25,26].

In this work, a kinetic model reported in the literature for POM oxidation is studied [27]. This kinetic model has been widely used for methane ATR modeling and simulation works. Firstly, the model was solved with the reported parameters and then the kinetic parameters were recalculated. The experimental data from the literature were used to recalculate the kinetic parameters. The predictions of product composition with values of both sets of parameters (reported and recalculated) exhibit different accuracy. The kinetic study, focused on temperature variations, is accurate for methane ATR modeling. Further studies regarding other operating conditions may confirm the proposed reaction scheme for the POM.

Although a kinetic model based on detailed reaction mechanisms can be used, we developed an intermediate kinetic model, neither too robust (microkinetic model) nor too simple (power-law model), which is represented by the LHHW mechanisms. It should be stated that the robustness of a kinetic model must be confirmed by proper predictions of experimental data, and our results show good accuracy for those predictions.

## 2. Methodology

### 2.1. Experimental Data Collection

**Figure 1:**
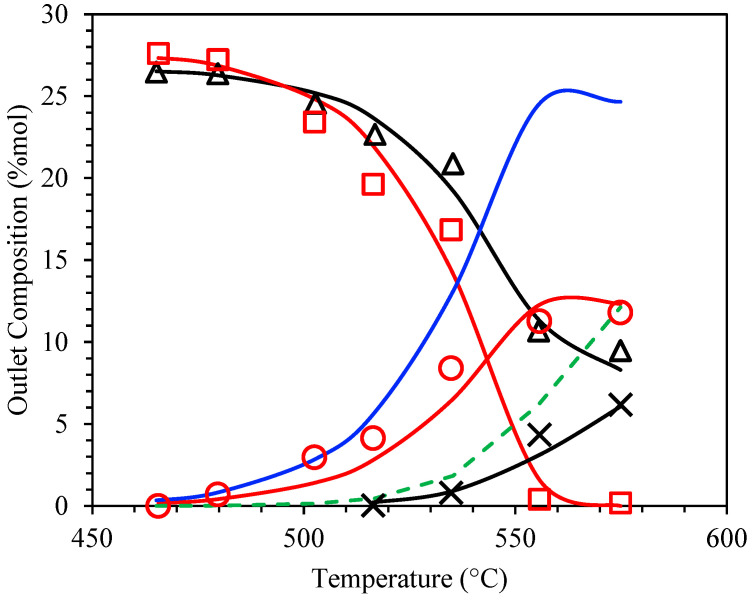
Experimental (symbols) and calculated by the proposed model (red and black lines) outlet compositions of (△) methane, (□) oxygen, (**○**) carbon dioxide, (**×**) carbon monoxide, (blue line) water, (green dashed line) hydrogen vs. operating temperatures. Molar feed composition: CH_4_ (26.7%), O_2_ (27.7%), carrier gas N_2_ (45.6%) at 1 atm, space time of 1377 $g_{cat}s/mol$.

The POM experimental data were taken from the literature [27] and used for the validation of the kinetic model. The tests were carried out in a packed-bed reactor (PBR) over 0.02–0.06 g of Pt/Al_2_O_3_ catalyst. The experimental data consist of different product compositions (CH_4_, O_2_, CO_2_, and CO) obtained in the range of operating temperatures of 465–574 °C at fixed space time of 1377 g_cat_s/mol. A stream with 26.7 mol% methane and 27.7 mol% oxygen was fed to the reactor; the remaining composition was composed of nitrogen as a carrier gas. The compositions of products were analyzed with chromatograph equipment. Figure 1 shows the profiles of experimental product compositions (symbols) with respect to the operating temperature. Predictions (lines) are also plotted, which will be discussed in following sections. A strong composition dependence for methane (triangles) and oxygen (squares) is observed for both profiles along the temperature range. That is methane content changes from 25 to 10 mol% while oxygen changes from 28 to 0.1 mol% when temperature increases from 465 to 575 °C. The conversions of methane and oxygen are low at 465 °C, but as the operating temperature is higher, both compositions decrease at a high rate and they stabilize at about 555 °C, which may be due to the depletion of oxygen. This indicates a strong dependence of methane composition on oxygen consumption. The difference in the slopes of methane and oxygen composition profiles is notable from 490 °C since oxygen is consumed in a greater proportion than methane according to the stoichiometric coefficients of reaction R1. The experimental data of methane and oxygen at 535 °C were slightly out of the decreasing tendency, but they deviated at the same proportion, which may be due to experimental errors or to operating issues during the tests. The dependence of methane on oxygen is confirmed at temperature > 550 °C at which both compound compositions have a similar abrupt drop. At 558 °C the oxygen composition is near zero, indicating almost total consumption of oxygen. In addition, the production of carbon monoxide is significantly increased at that temperature. The production of CO_2_ is observed at temperatures higher than 475 °C. From this value, the conversion of methane and oxygen becomes significant, indicating the relationship of CO_2_ composition on methane and oxygen consumption based on reaction R1. The increasing behavior of the carbon dioxide composition follows the decreasing tendency of the methane profile. In the range of 550–580 °C the production of carbon dioxide is slowed down due to the depletion of the oxygen reactant (R1). The CO_2_ profile cannot be completely dependent on methane since CO is a component produced by methane partial oxidation through reaction R2. The CO composition becomes significant when the concentration of oxygen is reduced at about 550 °C, which suggests that it is produced by the incomplete oxidation of methane. An increase in the production of carbon monoxide is observed in the range of 550–580 °C, where a small rise in the consumption of methane corresponds to an increase in carbon monoxide production, which indicates the promotion of incomplete methane oxidation. The inclusion of water compositions in the experimental data can be a key factor for the determination of the reaction schemes for POM. In this case in the literature, the composition of water and hydrogen at the exit was not reported in the experimental data.

### 2.2. Mathematical Modeling of Reaction Kinetics

The kinetic model in the literature considered the consumption of reactants (methane and oxygen) and generation of products (CO_2_ and CO) by reactions R1 and R2, since the molar concentrations of these components are available in the POM experimental data (Figure 1). The reported model from the literature is based on the LHHW mechanism in which the adsorbed methane reacts with both the adsorbed and gas-phase (Eley-Rideal) oxygen:

(6) $$r_{CH_{4}}^{*}=\frac{k_{1a}y_{CH_{4}}y_{O_{2}}}{{\left(1+y_{CH_{4}}K_{CH_{4}}+y_{O_{2}}K_{O_{2}}\right)}^{2}}+\frac{k_{1b}y_{CH_{4}}y_{O_{2}}^{1/2}}{1+y_{CH_{4}}K_{CH_{4}}+y_{O_{2}}K_{O_{2}}}$$

where $k_{1a}$ is the reaction rate coefficient for POM with a molecule of oxygen, $k_{1b}$ is the reaction rate coefficient for POM with half a molecule of oxygen, $y_{i}$ is the concentration of component i in mole fraction, and $K_{i}$ is the adsorption constant of component i. During kinetic modeling it was found that the experimental data of POM can be predicted by the reaction scheme composed of the reactions R1 and R2.

During parameter estimation, the calculated values of methane and product adsorption constants were obtained to be much lower than 1 with the Sequential Quadratic Programming (SQP) method, and the oxygen adsorption constant resulted to be the only significant one among the other components $\left(K_{CH_{4}},K_{CO_{2}},K_{CO},K_{H_{2}},\;K_{H_{2}O}\approx 0\right)$. Then, the corresponding kinetic equations based on the LHHW resulted as follows:

(7) $$r_{1}=\frac{k_{1}y_{CH_{4}}y_{O_{2}}}{{\left(1+y_{O_{2}}K_{O_{2}}\right)}^{2}}$$

(8) $$r_{2}=\frac{k_{2}y_{CH_{4}}y_{O_{2}}^{1/2}}{{\left(1+y_{O_{2}}K_{O_{2}}\right)}^{2}}$$

These reaction rate equations do not contain any equilibrium constant since POM reactions (R1 and R2) are irreversible. Equations (7) and (8) assume that the reactions (R1) and (R2) are carried out in the dual-site mechanism. The first term of Equation (7) indicates that the complete combustion of methane (R1) does not behave as an elemental reaction. The non-elemental order of the reaction in the rate equation indicates that the reaction is not carried out as homogeneous, but a still-undetermined underlying mechanism governs the rate of reaction (R1).

The negligible effect of water, carbon monoxide, and carbon dioxide on the reaction rates was reported experimentally in the model from the literature [27]. Some studies confirmed that the influence of steam, hydrogen, and carbon dioxide in the surface coverage of POM catalyst is negligible [18]. Competitive adsorption between methane, carbon dioxide, and water has been reported over the Rh catalyst [6,16,28]. But numerous conclusions have been pointed out in literature. The reaction rate Equations (7) and (8) consider that reactions (R1) and (R2) are carried out by the dual-site mechanism. The value of the reaction rate coefficient k and adsorption constant K are determined by the Arrhenius equation:

(9) $$k_{j}=A_{oj}\exp\left(-\frac{E_{Aj}}{R_{g}T}\right)$$

(10) $$K_{i}=A_{i}\exp\left(-\frac{{\Delta H}_{i}}{R_{g}T}\right)$$

where $k_{j}$ is the rate coefficient of reaction j, $K_{i}$ is the adsorption constant of component i, $A_{oj}$ is the preexponential factor of reaction j, $A_{oi}$ is the pre-exponential factor of adsorption of component i, $E_{Aj}$ is the activation energy of reaction j, ${\Delta H}_{i}$ is the energy of adsorption of component *i*, and $R_{g}$ is the ideal gas constant. The coefficients of the other components different than methane based on reactions R1 and R2 were not reported explicitly in the model from the literature according to experimental data. Therefore, they were assumed to be the following:

(11) $$\;\;\;\;\;\;\;\;\;\;\;\;\;\;\;r_{CH_{4}}=-r_{1}-r_{2}$$

(12) $$\;\;\;\;\;\;\;\;\;\;\;\;\;\;\;\;\;\;\;\;r_{O_{2}}=-2r_{1}-1/2r_{2}$$

(13) $$\;\;r_{CO_{2}}=r_{1}$$

(14) $$r_{CO}=r_{2}$$

Since SR experimental data commonly occurs after oxygen depletion, the experimental data at temperatures higher than 550 °C that can be attributed to SR reactions are few. Then, SR reactions were not considered in the proposed kinetic model [27]. It has been suggested that SR (R3, R4) and WGS (R5) reactions also contribute to carbon dioxide production, since water may be significant. However, further experimental studies should confirm this hypothesis, i.e., POM under oxygen-limiting concentrations.

### 2.3. Reactor Conservation Equations and Modeling

The mass balance equation of the experimental PBR is:

(15) $$\frac{dy_{CH_{4}}}{d\left(\frac{W}{F}\right)}=-r_{CH_{4}}$$

where $y_{CH_{4}}$ is the molar concentration of methane in mole fraction, $\frac{W}{F}$ is the space time of the total feed flow, and $r_{CH_{4}}$ is the rate of consumption of methane. According to the classification of Froment et al. [29], it corresponds to a 1-D pseudohomogeneous model. The initial conditions were set as follows:

(16) $$At\;z=0:\;\;\;y_{CH_{4}}=26.7\;mol\%\;and\;y_{O_{2}}=27.7\;mol\%$$

This equation was integrated with respect to $\frac{W}{F}$ using a Runge–Kutta integration method with a limit value of 1377 g_cat_s/mol. The operating temperature was set according to the experimental values reported by the authors. The final value for the integration of Equation (15) was the concentration at the outlet of the reactor, and it was compared with the corresponding experimental data. As reported in the model from the literature, diffusional effects were neglected by applying the Weiz and Mears criteria [30]. To validate the isothermal 1-D model, transport and thermal criteria for Trimm and Lam [27] reactor conditions were performed. Using the observed rate at 575 °C and space time of 1377 $\frac{g_{cat}s}{mol}\;$ Mears criterion was found to be significantly lower than 0.15, and the Weisz–Prater module remained below 1. This justifies neglecting mass transport limitations. Despite the exothermicity of the reaction, the small reactor diameter and the high surface-to-volume ratio ensure efficient heat dissipation. The calculated axial temperature deviation was minimal (lower than 5 K). The Weiz and Mears criteria were calculated with the following expressions.

(17) $$r_{obs}=\frac{X_{CH_{4}}}{\frac{W}{F}}$$

(18) $$\Phi_{WP}=\frac{r_{obs}\rho_{p}R^{2}}{D_{eff}C_{S}}$$

(19) $$C_{M}=\frac{r_{obs}\rho_{b}R}{k_{c}C_{b}}$$

where $\rho_{b}$ is the bulk density of the catalyst bed, R is the radius of the catalytic particle, $k_{c}$ the mass transfer coefficient, $C_{b}$ the methane concentration in the gas phase, and $D_{eff}$ is the effective diffusion in the catalytic particle. At the highest conversion experimental data (574 °C, $X_{CH_{4}}$= 0.645), the observed rate was $1.25\times \;10^{-4}\;mol/g_{cat}s$. Using this maximum rate, the Weisz–Prater criterion $\Phi_{WP}$ was found to be 0.12, which is two orders of magnitude below the unit threshold. Similarly, the Mears criterion ($C_{M}$ = 0.016) remained below 0.05.

### 2.4. Optimization Approach and Parameter Fitting

Parameter estimation was performed by the minimization of the objective function (OF) given by Equation (20), which is based on the average absolute error (AAE):

(20) $$OF=Min\left(AAE\right)=Min\left[\frac{\sum_{k=1}^{N_{exp}\;}\frac{\left|y_{i,calc}-y_{i,exp}\right|}{y_{i,exp}}}{N}\times 100\right]$$

where $y_{i,calc}$ is the calculated molar concentration of component i, $y_{i,exp}$ is the experimental molar concentration of component i, and N is the number of experimental data points. The objective function was minimized by using the SQP method. This method was used for parameter estimation since all the parameters are varied simultaneously in the search of the optimum set of values. The system of Ordinary Differential Equations (ODEs) was solved using the Runge–Kutta method. The optimization tolerance for the objective function was set to $10^{-6}$. The maximum values for the activation energies of reactions R1 and R2 were 30,000 to 150,000 J/mol and for the adsorption enthalpies were −20,000 to −150,000 J/mol based on Bischoff et al. [29].

The kinetic modeling procedure consisted of three steps of parameter estimation by SQL and Powel optimization methods:

1. Initial guesses. Since the kinetic parameters in the literature yielded deviated predictions, only the energies of activation and adsorption from the literature were kept as correct because they are between the typical values. Then, the pre-exponential factors of the Arrhenius equation were calculated.
2. Calculation of reaction rate coefficients. Once the Arrhenius parameters were defined, the values of the kinetic parameters were calculated. In this step the obtained values of parameters fitted the experimental data with good accuracy, but various parameters were lower than $1\times 10^{-15}$. For this reason, the adsorption parameters of all the components, except oxygen, were discarded from the denominator of the kinetic equations.
3. Sensitivity analysis. A sensitivity analysis was carried out to the obtained parameters to assure that they are the optimum ones. A variation of each parameter is made by maintaining the values of the other parameters to seek if a minimum value of the objective function is obtained. After this last confirmation, a statistical analysis was made to evaluate the predictions obtained with the optimum parameters.

## 3. Results and Discussion

### 3.1. Reproduction and Validation of Prior Kinetic Model

The kinetic model from the literature was reproduced to describe the experimental data reported by the authors [27]. The solution of the model in the literature was performed using the equations described in previous sections with the values of the parameters in the literature reported in the second column of Table 1. The product compositions calculated with the parameters in the literature strongly differed compared with the experimental data (not shown in Figure 1). The reason behind this poor prediction of compositions with the parameters in the literature is unknown. Reporting erroneous parameter values, their units, or mistakes during the programming of the differential equation and solution methods, could be some of the reasons.

### 3.2. Refinement of Kinetic Data

Due to the failed reproduction of the kinetic model with the parameters from the literature, a recalculation of parameters was carried out with the reaction rate Equations (7) and (8). The SQP method was used for the calculation of the parameters. During parameter estimation, multiple solutions were obtained, but in all cases the values of the adsorption constants of the species different than oxygen were near zero. This could be due to several combinations of numerator and denominator in Equations (7) and (8) that produce the same error in predictions. Then, this parameter was discarded from the kinetic model. With this consideration, the parameter estimation was reinitialized.

This suggests that the POM is carried out mostly in the catalytic phase, omitting the Eley-Rideal mechanism. The number of iterations of the SQP method was considerably reduced, mainly due to the reduced number of parameters. The parameters taken from the model from the literature and the recalculated ones in this work are shown in Table 1. The values of the recalculated parameters and the ones from the literature are significantly different regarding reaction rate coefficient and adsorption constants, while the difference in activation energy is not considerable, which was expected since the activation and adsorption energies should not change considerably due to the nature of the reactions. The obtained value of the activation energy for reaction R1 can be explained by the lower energy required to carry out this reaction. This value is similar to that obtained in the literature [31,32]. The absence of the methane adsorption constant suggests that the oxygen adsorption is considerably higher than that of methane which agrees with Osman et al. [7]. The calculated activation energy of 51,320 J/mol is characteristic of an adsorption-controlled regime (typically dominant between 400 and 600 °C), where high surface coverage leads to a lower activation energy compared with the higher intrinsic energies observed under differential conditions. Rather than a purely mathematical artifact, this comparison reflects the interplay between adsorption and reaction on the Ni surface. The activation energy of reaction R2 is justified by the significant effect of temperature at higher values, as observed in the concentration profiles of CO_2_ and CO for R1 and R2, respectively. The experimental (symbols) and calculated (lines) molar concentrations obtained with the optimized parameters are shown in Figure 1. The product composition profiles of the experimental data are accurately predicted by the proposed model. In the range of 554–575 °C, the calculated profiles show slight deviations for methane. Since oxygen is mostly consumed in the partial oxidation reaction, this indicates that it is the limiting reactant of the POM reactions. The CO_2_ profile was predicted by the model with no significant deviations. In the range of 550–580 °C, the CO_2_ profile was flattened where the oxygen concentration is low. The CO profile accurately predicted the experimental data in the range of temperature of 510–580 °C where the molar concentrations are significant. It has been proposed that SR reactions can be carried out due to the presence of the water produced by the POM. To confirm this, more detailed experimental information is necessary.

### 3.3. Statistical Analysis and Performance Evaluation

A statistical analysis was performed to evaluate the predictions of experimental data by the proposed model. The value of the obtained determination (R^2^) coefficient is 0.988, which indicates good predictability of the experimental data. The predictions of the experimental data of oxygen in the range of temperatures of 500–535 °C were the most deviated. Those deviations strongly impact on the value of R^2^. The deviations in the predictions were not considerable for the profile of CO_2_ concentration. The hold-up of the increasing molar composition of CO_2_ at temperatures higher than 550 °C was predicted by the kinetic equation of reaction R1. The increasing trend of the CO molar composition at temperatures higher than 510 °C was accurately predicted by reaction R2 of the kinetic model.

**Figure 2:**
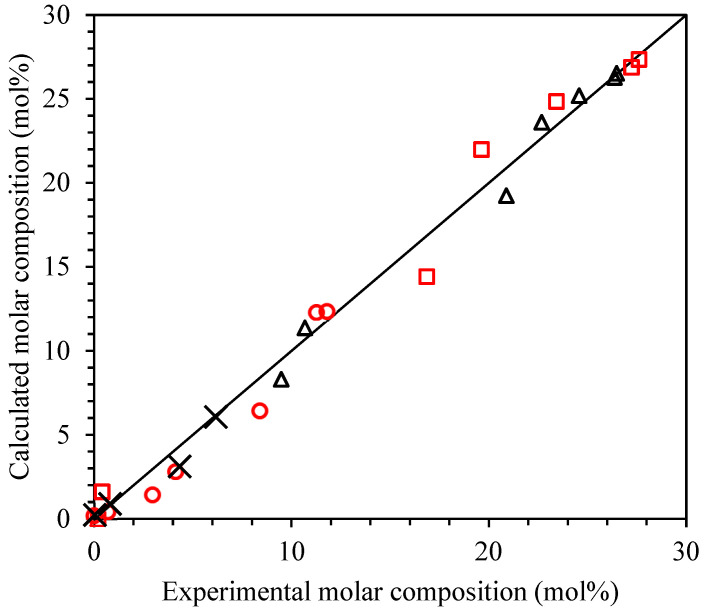
Parity plots of predictions of proposed model for (▲) methane, (■) oxygen, (**○**) carbon dioxide, and (**×**) carbon monoxide.

**Figure 3:**
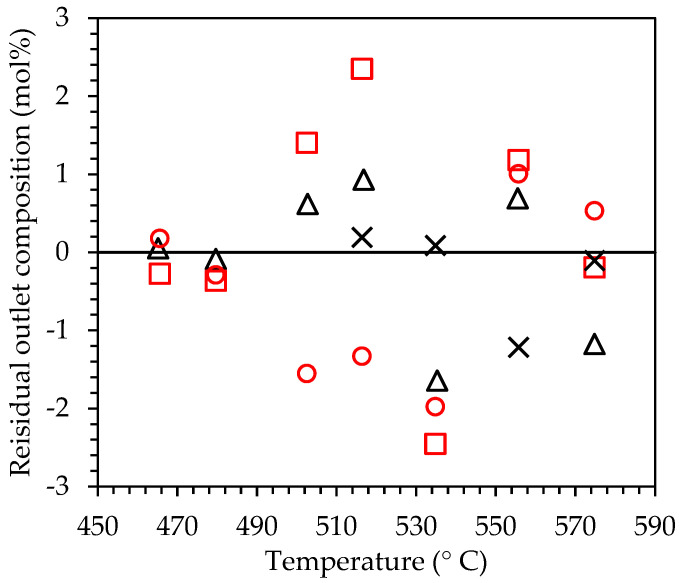
Residual plot of predictions of proposed model for (▲) methane, (■) oxygen, (**○**) carbon dioxide, and (**×**) carbon monoxide.

Figure 2 presents the parity plot of the experimental and calculated product compositions. The scatter of the points is lower for the lowest and highest compositions for methane and oxygen. The highest deviations are found in the range of molar compositions of 15–25 mol% for oxygen and methane. The model showed some deviations for methane, oxygen and CO at around 555 °C at which the highest changes in molar concentration with respect to temperature were registered. This confirms the difficulty of predicting experimental data at the highest temperatures at which the methane conversion is limited by the almost completely consumed oxygen. This observation is also seen in the residual plot (Figure 3) for the two highest operating temperatures.

From the parity plot and residual plot, various statistical parameters were obtained, which are reported in Table 2. The value of the parity-plot slope was 1.0179, which indicates that the calculated values were near experimental data. The low negative value of the intercept indicates the low deviation of the model predictions to the experimental data. The lowest and highest residual values are not considerable compared with the experimental compositions. The AAE% also presents a low value, which indicates good accuracy of the predictions. The values of the highest negative and positive residuals are accurate.

An advantage of the proposed model is that the number of parameters is lower compared with the kinetic model from literature, which suggests that the description of the experimental data by the kinetic model from literature is achieved through overfitting.

**Figure 4:**
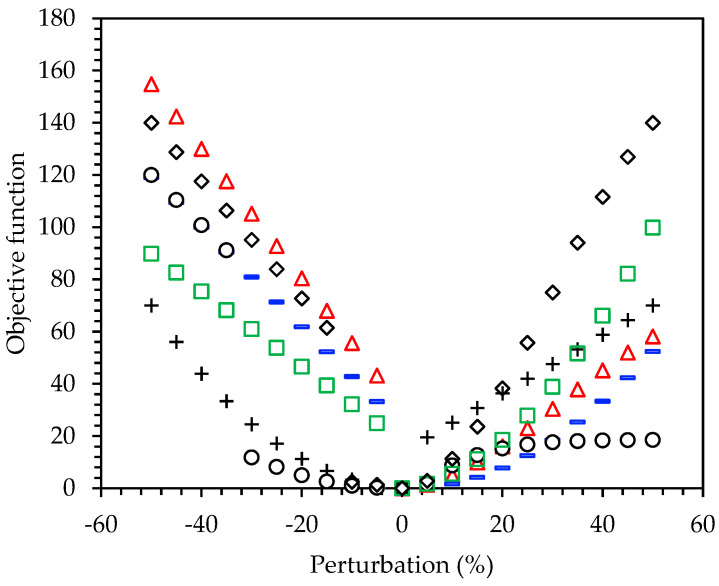
Sensitivity analysis of kinetic parameters of the proposed model. (+) $A_{oR1}$, (△)$E_{AR1}$, (**−**) $A_{oO_{2}}$, (○) $E_{AO_{2}}$, (□) $A_{oR_{2}}$, (◊) $E_{AR_{2}}$.

Figure 4 depicts the sensitivity analysis for the kinetic parameters of the proposed model. $E_{AR1}$ has the strongest effect on AAE due to the exponential increasing effect on the reaction rate coefficient $k_{R1}$. $A_{oR1}$ has one of the strongest effects on the objective function since it belongs to the parameter $k_{R1}$. The less sensible parameter was $A_{oO_{2}}$, which indicates that oxygen adsorption has a lighter effect than that of oxygen in the reaction rate equation. Tavazzi et al. [32] also reported disregarding the methane adsorption parameter, as their LHHW kinetic model for POM omitted the methane partial pressure term in the denominator. The sensitivity analysis for ${\Delta H}_{O_{2}}$ has a significantly higher effect on the objective function due to the exponential function. A local false minimum value of two parameters was found in the sensitivity analysis of ${\Delta H}_{O_{2}}$ and $A_{oO_{2}}$ at perturbations of 20%, but at higher perturbations the value of the objective function increased considerably. Regarding the remaining parameters, the expected behavior with the perturbation of the parameters was observed. AAE is highly sensitive to each parameter; even minor perturbations in the activation energy or the adsorption constants lead to a substantial increase in the residual error. This high sensitivity confirms that the parameters are statistically identifiable and that the optimization has achieved a well-defined global minimum. To address the concern regarding parameter robustness, a cross-validation study was performed by removing 30% of the intermediate temperature experimental data points. The resulting activation energy stabilized at 71,700 J/mol. The value of $E_{A1}$ indicates a physically significant value during cross-validation and not mathematical artifacts.

Table 3 presents the mass balance closure for the effluent components. The observed deviations are lower than 1% with respect to the feed. This strengthens the accuracy of the proposed kinetic model to the principle of mass conservation across the investigated operating range. The predicted experimental data were compared with other source of experimental data [32], which covers a broader temperature range. A qualitative agreement in the species profiles was observed. Oxygen consumption exhibited a similar dependence on methane when temperature is raised. The concentrations of $CO_{2}$ and $H_{2}O$ increased when the conversion of reactants (methane and oxygen) was almost completed through reaction R1. Conversely, the CO and $H_{2}$ concentrations increase significantly after oxygen is depleted, aligning with the subsequent partial oxidation. This behavior provides compelling evidence of the proposed reaction network carried out in the POM. It has been found that CO in the feed has an inhibiting effect on the methane conversion at temperatures higher than 600 °C [33]. Due to the scarce experimental data of CO, the effect of catalytic deactivation could not be clearly detected and predicted by the kinetic model. During the sensitivity analysis, it was found that adding parameters of CO (adsorption constant) had a negligible effect on POM performance. This might be due to the low concentration of CO and the presence of the other species. It was also reported that the presence of oxygen during POM performance promoted the oxidation and release of CO from the catalyst [33]. The presence of water produced by reaction (R1) can promote SR reactions (R3)–(R5). However, the experimental data at which SR reactions can participate is scarce in Figure 1. Then, for this work, reactions (R3)–(R5) had to be neglected from the kinetic modeling. Furthermore, experimental data of hydrogen or water must be available to investigate the participation of SR reactions.

### 3.4. Model Applications and Limitations

All the previous results demonstrate the good accuracy of the proposed kinetic model with recalculated parameters and the preciseness of the parameter estimation procedure used to accurately describe the POM reaction. This scheme of reactions (R1) and (R2) keep the ratios of CO/H_2_ and CO_2_/H_2_O constant in the products along the predicted range of temperature (450–600 °C). This represents a deviation from the real behavior of POM when the operating conditions promote the depletion of oxygen, i.e., higher temperatures, low inlet oxygen composition, or significant inlet compositions of water, since SR reactions become competitive. However, this kinetic model focuses on methane oxidation reactions, which are currently discussed in the literature about which of them are carried out in the POM (direct or indirect pathway). The significance of this work is supported mainly by the prediction of POM experimental data at significant oxygen compositions, i.e., low methane and oxygen conversions. Accurate predictions may be carried out in the range of 450–600 °C in the POM at significant concentrations of oxygen. The discard of methane adsorption coefficient of the model from the literature implied less time for parameter estimation, while the difference in predictability between the literature and proposed model was small. There is still uncertainty about the reaction scheme for the POM, which represents a matter in kinetic modeling. The availability of experimental water compositions can be a key for finding the remaining reaction. Not only should the reaction schemes in kinetic modeling be determined through mass balance, but also with energy balance. This suggests that the calculation of heat of reaction can be made through experimental temperature profiles along the catalyst bed. The activation energies of the proposed model and the one from the literature are not considerably different, which confirms the behavior of the rate of POM reactions with respect to temperature. The values of the adsorption energies of oxygen were similar. The negative values of oxygen adsorption energies confirm their behavior with temperature.

Although the controversy about the mechanism for the POM is not determined yet, some similitudes are found between different metal promoters (Ni, Rh, Pt, Pd), i.e., total oxidation of methane is carried out in various POM catalysts due to the detection of hot-spot temperature. Direct production of syngas through the partial oxidation of methane has been obtained at high values of GHSV or low concentrations of oxygen, which suggests that the direct pathway succeeds when the interaction between methane and oxygen is highly limited during catalytic POM performances. There has been continuous discussion about proving the two main reaction pathways. The vast information on POM experiments over a significant number of different catalysts comprises particular and apparently isolated cases. As mentioned above, plentiful experimental data containing POM results should be generated at wide ranges of temperatures, GHSV and inlet compositions.

Modeling the experimental data of POM is useful in the prediction of the first section of methane ATR. The experimental data of POM carried out at significant concentrations of oxygen depicts a low influence of SR reactions which allows the modeling of only the reactions (R1) and (R2). The calculation of the rates of (R1) and (R2) is useful in the prediction of the heat production and the profile of temperature along the catalyst bed of the ATR reactor. It is important to mention that the entire prediction of the outlet concentration of components and the temperature profile in methane ATR has not been achieved. The modeling of the POM experimental data over Pt/Al_2_O_3_ catalyst used can provide insights into general trends in POM reactions for Pt-supported and, if possible, in other types of catalysts, since Pt catalysts offer the greatest performance in POM. Further experiments over other catalysts can support the trends found in this work. The proposed kinetic model for POM over Pt/Al_2_O_3_ can be extended for Pt catalysts over other supports with the same parameters or they can be recalculated if the necessary data are available. The relationship between methane and oxygen reactivities for reactions R1 and R2 was also confirmed. The predicted experimental data can be considered reliable for kinetic modeling purposes for the wide range of operating temperatures at which very low to significant conversions were registered. The proposed kinetic model demonstrates robust applicability to the experimental system under the specific conditions detailed in Section 2.1, particularly within the investigated temperature range at atmospheric pressure. While the current parameter set provides high statistical identifiability for this regime, further experimental efforts (incorporating broader feed compositions and elevated temperature ranges) are required to extend the model predictive capacity and ensure its generalization across different operating conditions.

## 4. Conclusions

From the kinetic modeling study, the following conclusions can be pointed out:

- The proposed model accurately describes experimental data of methane partial oxidation according to the values of the correlation coefficient and AAE. The proposed model can also predict the effect of temperature on the rates of methane partial oxidation reaction.
- The reproduction of the kinetic model in the literature did not achieve the reported results of product composition. The differences in the predictions may be explained by the method of optimization of the kinetic parameters.
- The generation of experimental data of methane partial oxidation for the study of the products with respect to temperature and space time can give great advance in the definition of the reaction schemes. The consideration of both the compositions and the temperature profiles in the catalyst bed can model the system and reduce bias for the scaling-up of the simulations.
- Experimental data for kinetic modeling should contain information that permits the study of the behavior of POM with respect to the main operating conditions at wide ranges (temperature, GHSV, feed composition, type of catalyst, etc.) since it has been found that the reaction scheme can change from one value of temperature to another, and according to GHSV, the concentration of oxygen, catalyst deactivation, etc.
- The deactivating effect of CO at temperatures below 600 °C could not be detected in this kinetic study due to the scarce experimental data of CO concentration in the products.
- The relationship between oxygen and methane along the range of operating temperatures was predicted by the reaction scheme of complete combustion of methane (R1) and partial oxidation of methane (R2).

## Figures and Tables

**Table 1:** Values of optimized parameters and parameters from the literature.

Parameter	Literature Model	This Work	Units
$k_{1}$	3.14 × 10^7^	0.585	$mol/g_{cat}s$
$E_{A1}$	86,000	41,620	J/mol
$k_{2}$	2.64 × 10^7^	32.641	$mol/g_{cat}s$
$E_{A2}$	86,000	145,655	J/mol
$K_{CH_{4}}$	6.67 × 10^2^	-	%mol
${\Delta H}_{CH_{4}}$	−27.3	-	J/mol
$K_{O_{2}}$	4.34 × 10^−5^	0.95442	%mol
${\Delta H}_{O_{2}}$	−92.8	−36,306	J/mol

K*_i_* and k*_i_* at 557 °C.

**Table 2:** Statistical results of the predictions with optimized parameters.

Parameter	Proposed Model
R^2^	0.988
Slope	1.0179
Intercept	−0.355
AAE (%)	0.324
(+) residual	13
(−) residual	12
Residual balance	−1
Lowest negative residual	−2.451
Highest negative residual	2.349
Range	4.08

**Table 3:** Mass balances.

T (°C)	$CH_{4}\left(\frac{g}{s}\right)$	$O_{2}\left(\frac{g}{s}\right)$	$CO_{2}\left(\frac{g}{s}\right)$	$H_{2}\left(\frac{g}{s}\right)$	$CO\left(\frac{g}{s}\right)$	$H_{2}O\left(\frac{g}{s}\right)$	$N_{2}\left(\frac{g}{s}\right)$	Total $\left(\frac{g}{s}\right)$
465.15	4.27	8.86	0.00	0.00	0.00	0.00	12.77	25.90
479.65	4.27	8.85	0.01	0.00	0.00	0.01	12.77	25.90
502.73	4.22	8.66	0.14	0.00	0.00	0.11	12.77	25.90
516.82	4.03	7.92	0.64	0.00	0.02	0.53	12.77	25.90
535.26	3.10	4.47	2.95	0.01	0.17	2.41	12.77	25.89
555.49	1.91	1.02	5.02	0.07	0.94	4.11	12.77	25.84
574.84	1.36	0.27	5.21	0.13	1.78	4.26	12.77	25.78

## Data Availability

The original contributions presented in this study are included in the article. Further inquiries can be directed to the corresponding author.

## Abbreviations

The following abbreviations are used in this manuscript:

AAE	Absolute average error
ATR	Autothermal reforming
POM	Catalytic partial oxidation
PBR	Packed-bed reactor
WGS	Water–gas shift reaction
$A_{0j}$	Pre-exponential factor of reaction *j*, $\left[\frac{mol}{g_{cat}s}\right]$
$A_{i}$	Pre-exponential factor for component *i*, %mol
$C_{b}$	Bulk concentration in the gas phase
$D_{eff}$	Effective diffusion in the catalytic particle $m^{2}/s$
$E_{Aj}$	Activation energy of reaction j, J/mol
$k_{j}$	Reaction rate coefficient of reaction *j*, $\left[\frac{mol}{g_{cat}s}\right]$
$K_{i}\;$	Absorption constant of component i
N	Number of experimental data
$R_{g}$	Ideal gas constant, J/(mol·K)
R	Radius of the catalytic particle
$r_{i}$	Rate of consumption of production of component i, $mol/(g_{cat}\cdot s)$
$r_{j}$	Rate of reaction *j*, $mol/(g_{cat}\cdot s)$
$r_{iAds}$	Rate of adsorption of component i, $mol/(g_{cat}\cdot s)$
T	Temperature, K
$\frac{W}{F}$	Space time of total feed flow, $\left[\frac{g_{cat}s}{mol}\right]$
$X_{i}$	Total conversion of component i
$y_{i}$	Outlet concentration of component *i*, (mole fraction)
$y_{i}^{*}$	Concentration of the component i in the catalyst surface, (mole fraction)
z	Axial length coordinate, m
$\Delta H_{j}$	Energy of absorption of component *j*, (J/mol)
$\Delta H_{298}$	Enthalpy of reaction at 298 K (J/mol)
$\rho_{b}$	Density of the catalyst bed
i	Component designation number
j	Number of reaction
R	Reaction
